# Supplementary material for: Hemidesmosomes and Notch signaling regulate epidermal differentiation via delamination
Source: Development. 2026 Apr 16;153(16):dev205210. doi: 10.1242/dev.205210 (PMC13120672; doi:10.1242/dev.205210)
Supplement: Supplementary information [file develop-153-205210-s1.pdf]

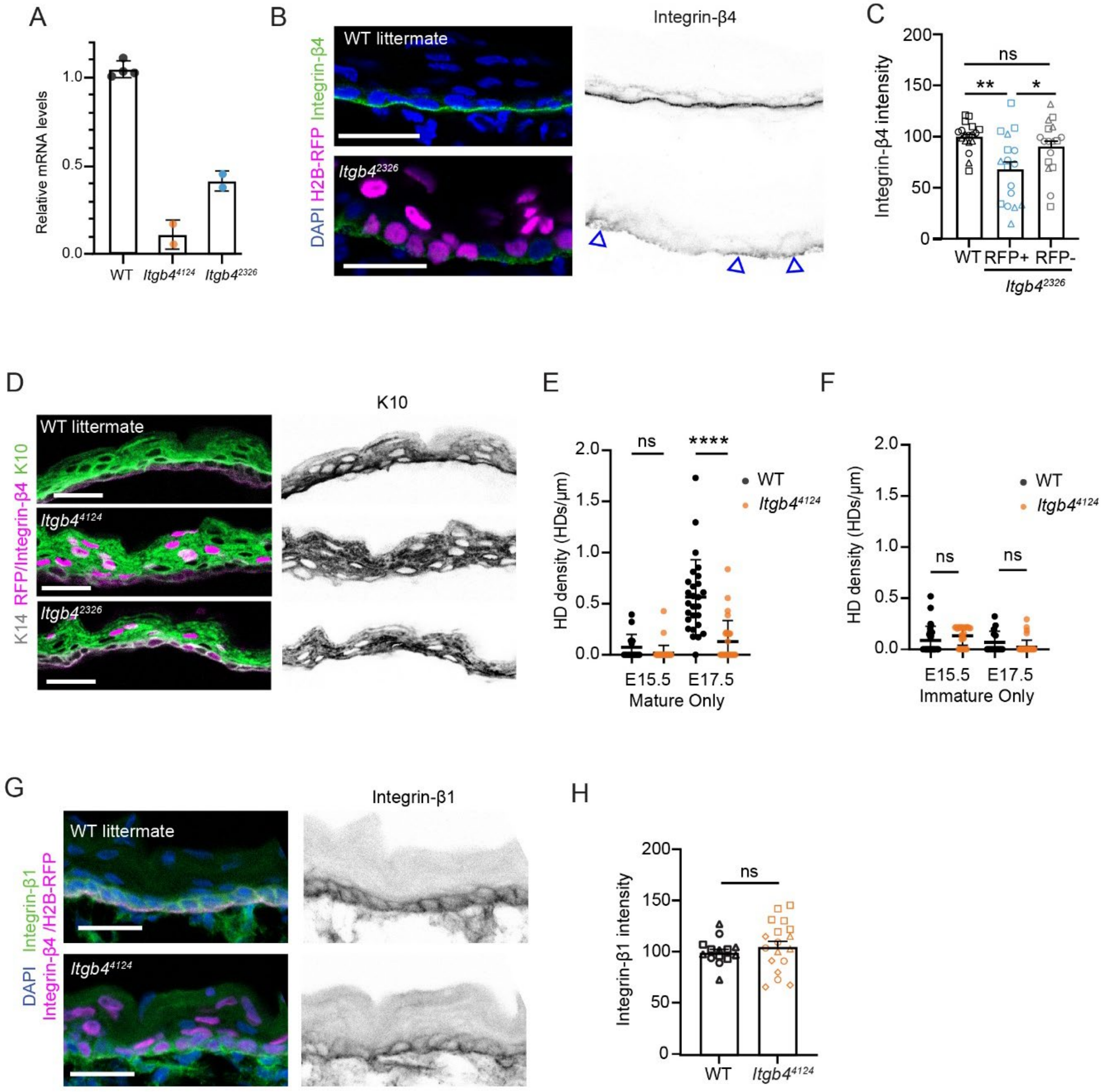

Fig. S1. (A) Relative mRNA abundance of *Itgb4* transcripts from *Scramble*, *Itgb4*^{4124}, and *Itgb4*^{2326} infected, puromycin-selected primary keratinocytes. mRNA levels normalized to *Scramble* per technical replicate. (B-C) Single-plane confocal images of E17.5 WT and *Itgb4*^{2326} infected epidermis showing RFP+ knockdown cells (magenta) and integrin-β4 (green) (B), with quantification of integrin-β4 fluorescent intensity (C). 6μm sum-slice confocal images of WT, *Itgb4*^{4124}, and *Itgb4*^{2326} infected epidermis showing cytokeratin-10 (K10, green) thickness. (E-F) Quantification of mature only (E) and immature only (F) hemidesmosome (HD) density per WT and *Itgb4*^{4124} nape skin. (G-H) Single-plane confocal images of E17.5 WT and *Itgb4*^{4124} infected epidermis showing integrin-β1 (green) (G) with quantification of intensity at DEJ (H).

Development • Supplementary information

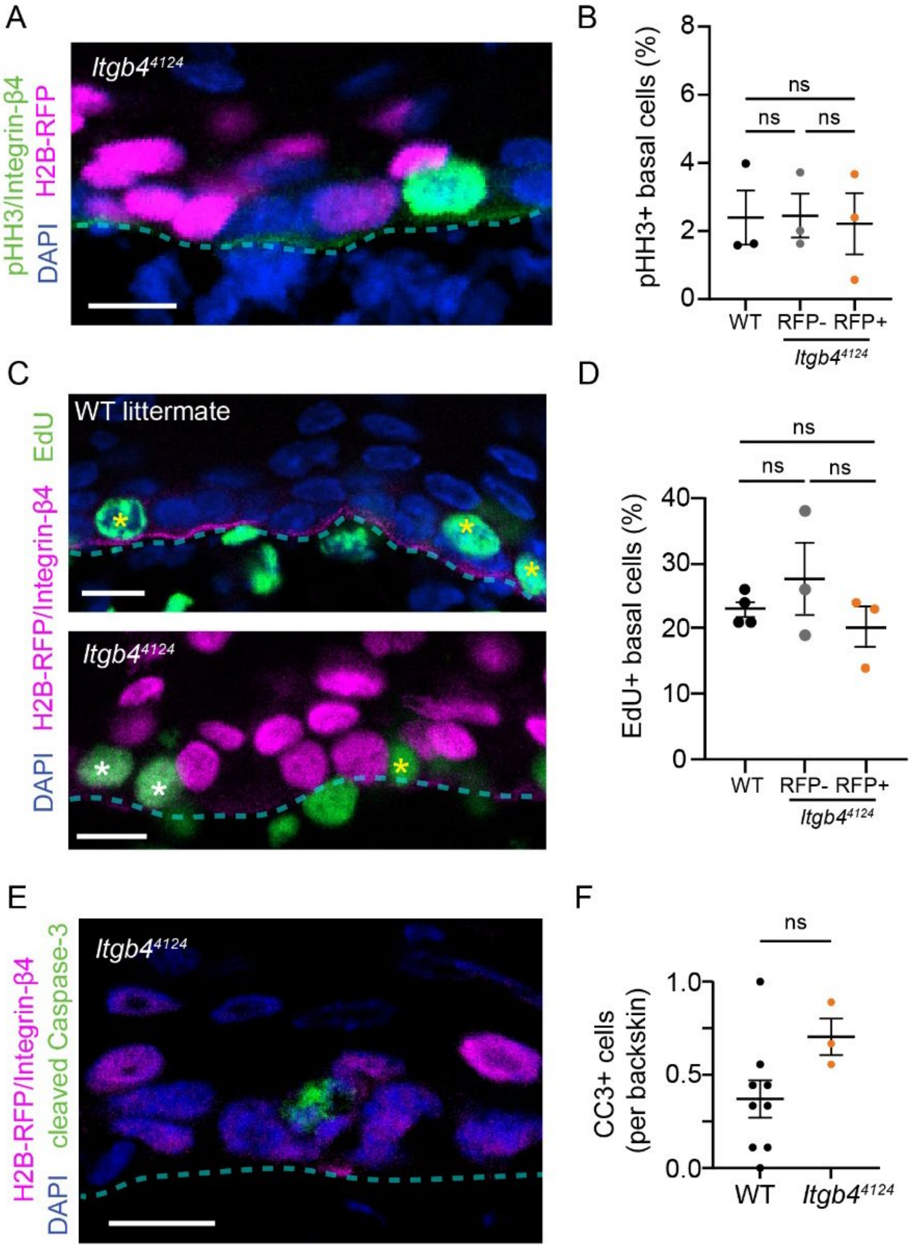

Fig. S2. (A-B) Single-plane confocal images of E17.5 WT and *Itgb4*^{4124} showing phosphor-histone H3 (pHH3, green) positive basal cells (A) with quantification (B) of total percentage of pHH3 cells in WT, RFP- and RFP+. (C-D) Single-plane confocal images of E17.5 WT and *Itgb4*^{4124} showing 5-ethynyl-2'-deoxyuridine (EdU, green) basal cells (C) with quantification (D) of total percentage of EdU+ cells in WT, RFP- and RFP+. Yellow asterisks indicate WT or RFP-, EdU+ basal cells. White asterisks represent RFP+, EdU+ basal cells. (E-F) Single-plane confocal images of E17.5 WT and *Itgb4*^{4124} showing cleaved caspase-3 (CC3, green) basal cells (E) with quantification (F) of total percentage of CC3+ basal cells per animal. Basement membrane is indicated with the dotted line (cyan) in (A, C, E). In (B, D, F) each dot represents the total percentage of cells per condition, per animal. Scale bars: 25μm in (A, C, E); p-values: ns, not significant.

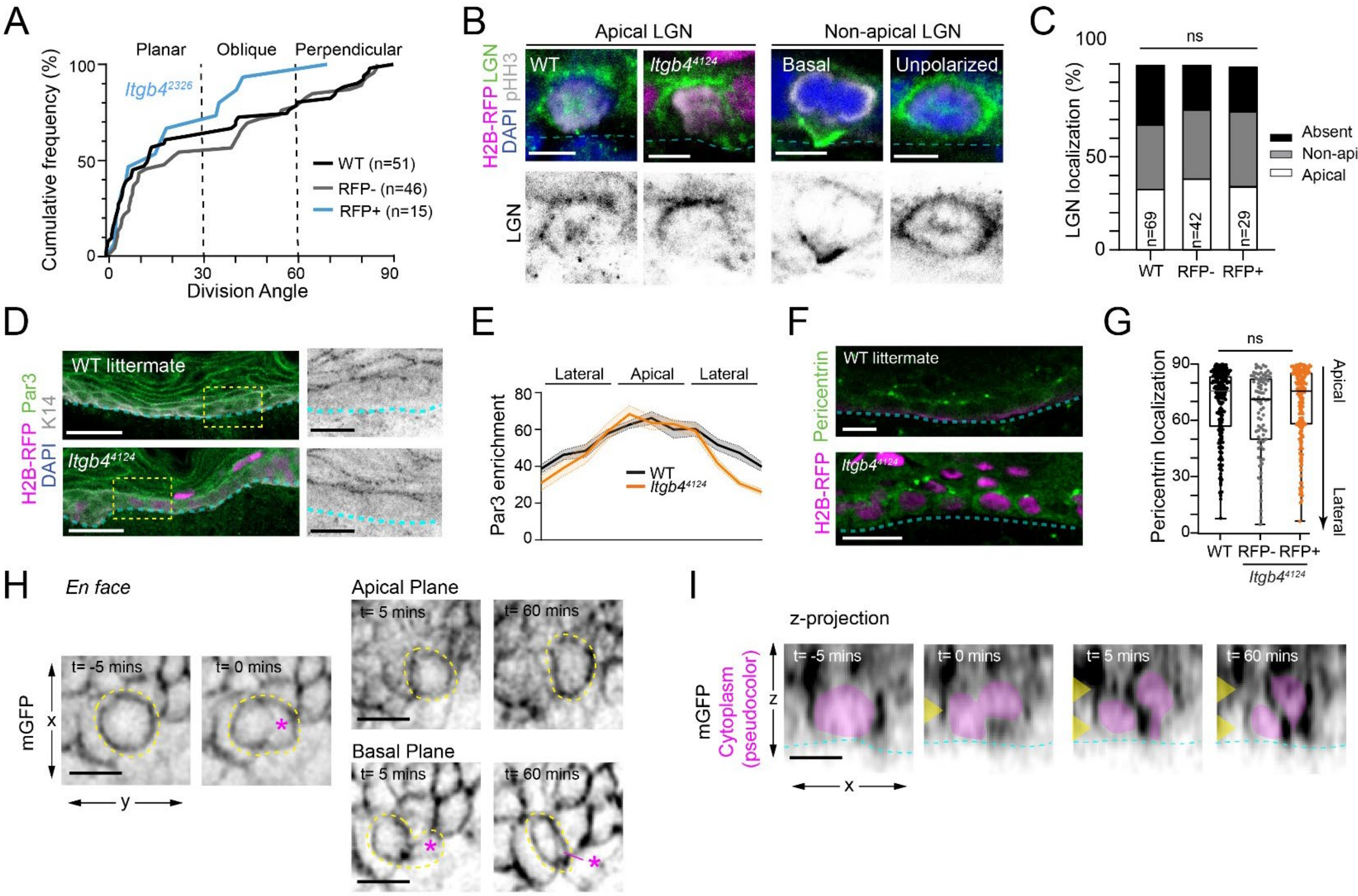

Fig. S3. (A) Cumulative frequency distribution of division angles for *Itgb4*^{2326} in WT, RFP-, and RFP+ basal cells. (B) Representative images of LGN (green) crescents in mitotic (pHH3), basal cells. (C) Distribution of LGN localization in all pHH3 positive cells per WT, RFP-, and RFP+. (D-E) Single-plane images of WT and *Itgb4*^{4124} epidermis showing Par3 (green) localization (D) with quantification (E) of cortical enrichment in WT and *Itgb4*^{4124} infected cells. (F-G) Representative images of WT and *Itgb4*^{4124} epidermis showing WT and RFP+ (magenta) basal cells with pericentrin (green) (F) with quantification (G) of the angle of the pericentrin puncta relative to the basement membrane in WT, RFP- and RFP+ cells. Note that in areas of integrin-β4 depletion, the basal surface of cytokeratin-14 (K14) or laminin-β3 was used to measure the angle of the basement membrane. (H-I) Representative timelapse images of an initial oblique-oriented cell with persistent basal endfoot where (H) is the *en face* view and (I) is the z-projection reconstruction. Apical and basal optical sections in (H) correspond with yellow arrows in (I). Magenta asterisks in (H) indicate the footprint of the daughter cell. Basement membrane is indicated with the dotted line (cyan) in (B, D, F, I). p-values: ns, not significant; **** p<0.0001 by observed vs. expected chi-square test in (D-F). Scale bars: 25µm in (D, F); 10µm in (B, H, I).

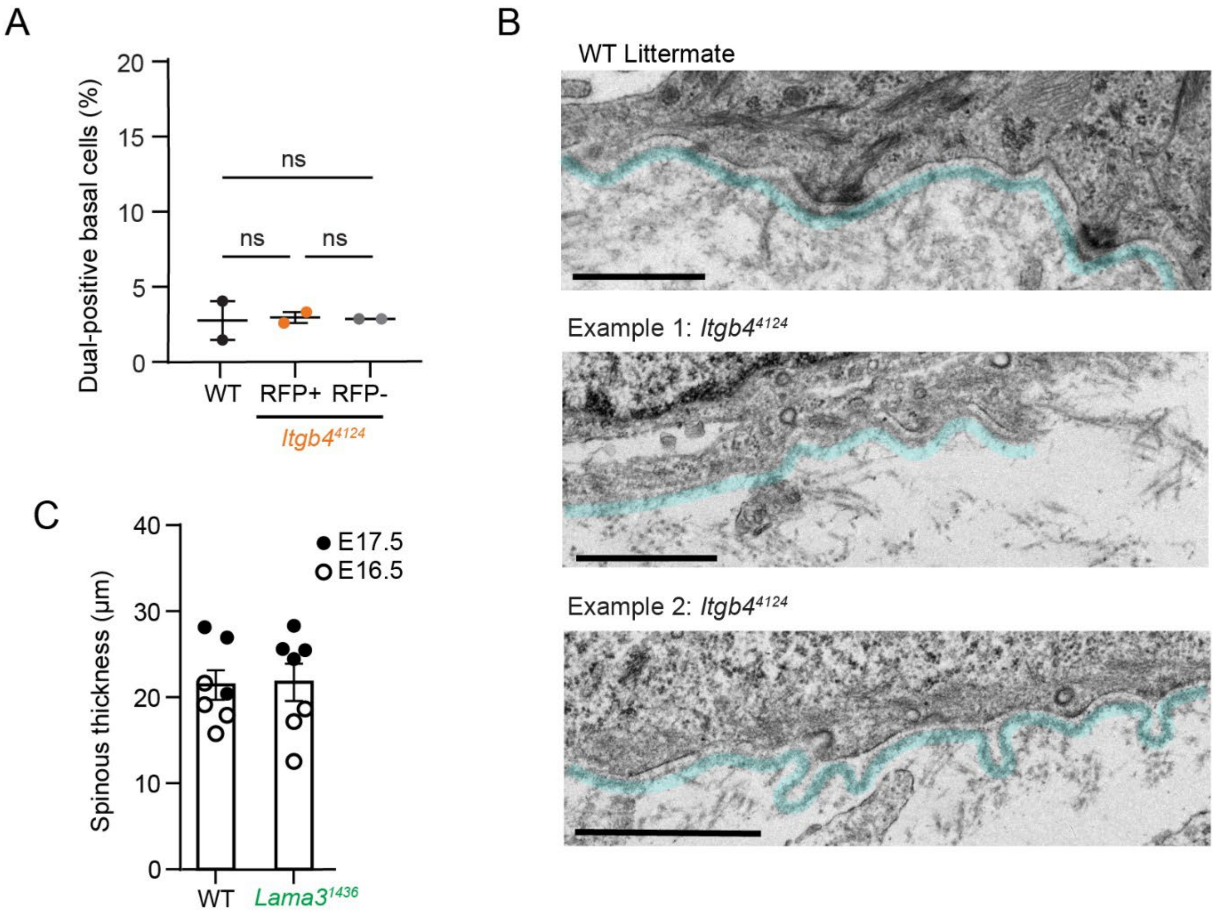

Fig. S4. (A) Quantification of the percentage of dual positive WT and *Itgb4*^{4124} (RFP- and RFP +) basal cells in E15.5 epidermis. (B) TEM micrograph of WT and *Itgb4*^{4124} mutant epidermis with aberrant laminae densa architecture (pseudocolored in cyan). (C) Quantification of cytokeratin-10 (K10), spinous thickness in all *Lama3*^{1436} mutant animals. Note that each animal is represented as a dot, and the age of the animal is indicated with an open (E16.5) or closed (E17.5) circle.

Development • Supplementary information

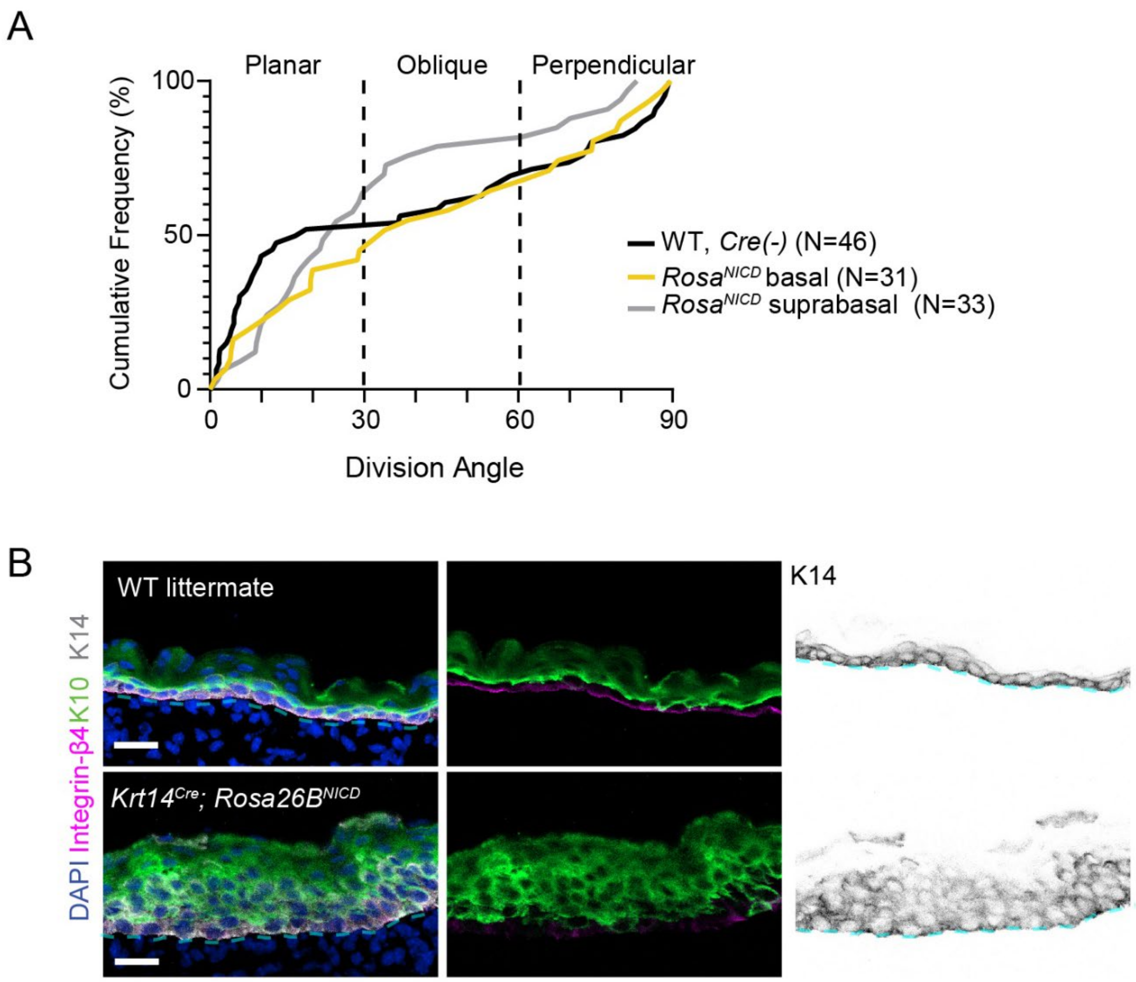

Fig. S5. (A) Cumulative frequency distribution of division angles in WT *Cre*-negative basal cells (black) versus *Krt14^{Cre};Rosa^{NICD}* basal (yellow) and suprabasal (grey) cells. Note that there were no suprabasal divisions in the *Cre*-negative, control littermates. (B) Single-plane confocal images of WT *Cre*-negative and *Krt14^{Cre};Rosa^{NICD}* epidermis showing cytokeratin-10 (K10, green) and single channel cytokeratin-14 (grey).

Development • Supplementary information
